# Antimicrobial Peptides

**DOI:** 10.3390/ph6121543

**Published:** 2013-11-28

**Authors:** Ali Adem Bahar, Dacheng Ren

**Affiliations:** 1 Department of Biomedical and Chemical Engineering, Syracuse University, Syracuse, NY 13244, USA; E-Mail: abahar@syr.edu; 2 Syracuse Biomaterials Institute, Syracuse University, Syracuse, NY 13244, USA; 3 Department of Civil and Environmental Engineering, Syracuse University, Syracuse, NY 13244, USA; 4 Department of Biology, Syracuse University, Syracuse, NY 13244, USA

**Keywords:** antimicrobial peptide, biofilm, persister

## Abstract

The rapid increase in drug-resistant infections has presented a serious challenge to antimicrobial therapies. The failure of the most potent antibiotics to kill “superbugs” emphasizes the urgent need to develop other control agents. Here we review the history and new development of antimicrobial peptides (AMPs), a growing class of natural and synthetic peptides with a wide spectrum of targets including viruses, bacteria, fungi, and parasites. We summarize the major types of AMPs, their modes of action, and the common mechanisms of AMP resistance. In addition, we discuss the principles for designing effective AMPs and the potential of using AMPs to control biofilms (multicellular structures of bacteria embedded in extracellular matrixes) and persister cells (dormant phenotypic variants of bacterial cells that are highly tolerant to antibiotics).

## 1. Sources and History of Antimicrobial Peptides

Antimicrobial peptides (AMPs) are oligopeptides with a varying number (from five to over a hundred) of amino acids. AMPs have a broad spectrum of targeted organisms ranging from viruses to parasites. Historically AMPs have also been referred to as cationic host defense peptides [1], anionic antimicrobial peptides/proteins [2], cationic amphipathic peptides [3], cationic AMPs [4], host defense peptides [5], and α-helical antimicrobial peptides [6].

The discovery of AMPs dates back to 1939, when Dubos [7,8] extracted an antimicrobial agent from a soil *Bacillus* strain. This extract was demonstrated to protect mice from pneumococci infection. In the following year, Hotchkiss and Dubos [9] fractionated this extract and identified an AMP which was named gramicidin. Despite some reported toxicity associated with intraperitoneal application [9], gramicidin was found effective for topical treatment of wounds and ulcers [10]. In 1941, another AMP, tyrocidine, was discovered and found to be effective against both Gram-negative and Gram-positive bacteria [11]. However, tyrocidine exhibited toxicity to human blood cells [12]. In the same year, another AMP was isolated from a plant *Triticumaestivum* [13], which was later named purothionin and found effective against fungi and some pathogenic bacteria [14].

The first reported animal-originated AMP is defensin, which was isolated from rabbit leukocytes in 1956 [15]. In the following years, bombinin from epithelia [16] and lactoferrin from cow milk [17] were both described. During the same time, it was also proven that human leukocytes contain AMPs in their lysosomes [18].

In total, more than 5,000 AMPs have been discovered or synthesized up to date [19]. Natural AMPs can be found in both prokaryotes (e.g., bacteria) and eukaryotes (e.g., protozoan, fungi, plants, insects, and animals) [20,21,22,23]. In animals, AMPs are mostly found in the tissues and organs that are exposed to airborne pathogens; and are believed to be the first line of the innate immune defense [24,25] against viruses, bacteria, and fungi [21]. Thus, AMPs play an important role in stopping most infections before they cause any symptoms. For example, frog skin is the source of more than 300 different AMPs [20,26].

Most AMPs are produced by specific cells at all times, while the production of some AMPs is inducible. For example, using silk moth as a model system, Hultmark and colleagues [27] demonstrated that P9A and P9B can be induced in hemolymph by vaccination with *Enterobacter cloacae*. In another study [28], epithelial cells from different tissues of mice showed increased rate of mRNA transcription for defensin production after infection with *Pseudomonas aeruginosa* PAO1.

Several types of eukaryotic cells are involved in AMP production such as lymphs, epithelial cells in gastrointestinal and genitourinary systems [29,30], phagocytes [31], and lymphocytes of the immune system [21,32]. In addition to direct involvement in innate immunity, AMPs have also been found to influence host’s inflammatory responses during an infection [33,34,35]. It is known that lipopolysaccharide (LPS) molecules, released from bacteria as a result of antibiotic treatment or host immunity, can induce AMP production in mammals [31]. For example, HEK293 cells produce defensin in response to LPS stimulation [36]. Some AMPs (e.g., CAP18 [37], CAP35 [38], and a lactoferrin-derivative [39]) can also block LPS-induced cytokine release by macrophages. Thus, these AMPs can reduce inflammatory response. In comparison, antibiotics do not have this type of regulation on inflammatory response of the host immune system; and LPS secretion following antibiotic treatment might cause over-reaction of the host immune system. In some extreme cases, this can even lead to sepsis [31,40].

## 2. Structure and Major Activities of AMPs

**Figure 1:**
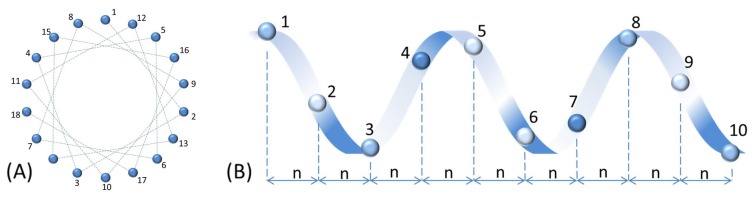
Schematic representation of an α-helical AMP. This figure assumes the same α-helix propensity for all amino acids in the peptide structure. (**A**) Helical wheel projection of the AMP (top view). The angle between two consecutive amino acids in the sequence is 100 degree. Dotted lines show two adjacent amino acids in the primary structure. (**B**) Side view of the peptide. The distance between two adjacent amino acids, “n”, is 0.15 nm.

Most AMPs reported to date can be characterized as one of the following four types based on their secondary structures: β-sheet, α-helix, extended, and loop. Among these structural groups, α-helix and β-sheet structures are more common [41]; and α-helical peptides are the most studied AMPs to date. In α-helix structures the distance between two adjacent amino acids is around 0.15 nm and the angle between them with regard to the center is around 100 degree from the top view (Figure 1A). The best known examples of such AMPs are protegrin, magainin, cyclic indolicin, and coiled indolicin [6]. β-sheet peptides are composed of at least two β-strands with disulfide bonds between these strands [42].

While most AMPs belong to one of the above four classes, some AMPs do not belong to any of these groups [43]. Some AMPs contain two different structural components [44]. Also, many peptides form their active structure only when they interact with the membranes of target cells. For example, indolicin shows globular and amphipathic conformation in aqueous solutions while it is wedge-shaped in lipid bilayer mimicking environments [45]. This AMP also changes its conformation during interaction with DNA evidenced with decreased fluorescence intensity and a slight shift in the wavelength of maximum emission [46].

Unlikely antibiotics, which target specific cellular activities (e.g., synthesis of DNA, protein, or cell wall), AMPs target the lipopolysaccharide layer of cell membrane, which is ubiquitous in microorganisms. Having a high level of cholesterol and low anionic charge puts eukaryotic cells out of the target range of many AMPs [47].

Another important feature of AMPs is their rapid killing effect. Some AMPs can kill in seconds after the initial contact with cell membrane [48]. AMPs are also known to enhance the activities of antibiotics through synergistic effects. For example, the combination of penicillin with pediocin and ampicillin with nisin Z exhibited killing of *Pseudomonas fluorescens* with 13- and 155-fold lower minimum inhibitory concentration (MIC), respectively, compared to using antibiotics alone [49].

Because AMPs are made with amino acids, it is relatively easy to modify the structure (including library construction and screening) and immobilize AMPs on surfaces [50]. It is possible to make fully synthetic peptides by chemical synthesis [51] or by using recombinant expression systems [52,53]. These artificial sources of AMPs are useful for modification of existing AMPs and for designing new synthetic AMPs. Such modifications have potential to change the targets of AMPs and improve the stability of AMPs against proteases [54].

Despite these advantageous features of AMPs, there are still some challenges to their applications, such as potential toxicity to humans [12,55,56], sensitivity to harsh environmental conditions (susceptibility to proteases and extreme pH [57,58]), lack of selectivity against specific strains [59], high production costs [60], folding issues of some large AMPs [61], reduced activity when used for surface coating [62], and bacterial resistance to some AMPs [63,64]. In the following section we will discuss the modes of actions of AMPs and the current efforts to address the above challenges.

## 3. Major Categories of AMPs and Mechanisms of Action

### 3.1. Classification

In general, enzymatic mechanisms are not involved in the antimicrobial activities of AMPs [65]. For example, even though lysozyme is a monomeric peptide, it is not classified as an AMP because it is relatively large (148 aa) and kills bacteria through enzymatic activities by breaking 1,4-β-linkages in peptidoglycan chains [66]. In this review, we categorize AMPs based on their target, and mode of action. For natural AMPs, we will focus on those from eukaryotes, especially mammals.

#### 3.1.1. Antiviral Peptides

Antiviral AMPs neutralize viruses by integrating in either the viral envelope or the host cell membrane. Previous studies have shown that both enveloped RNA and DNA viruses can be targeted by antiviral AMPs [67,68]. AMPs can integrate into viral envelopes and cause membrane instability, rendering the viruses unable to infect host cells [69,70]. AMPs can also reduce the binding of viruses to host cells [71]. For example, defensins bind to the viral glycoproteins making herpes simplex viruses (HSV) unable to bind to the surface of host cells [72].

Besides disruption of viral envelopes and blocking viral receptors, some antiviral AMPs can prevent viral particles from entering host cells by occupying specific receptors on mammalian cells [73,74]. For example, heparan sulfate is important for the attachment of HSV viral particles to the host cell surface [75]. The heparan sulfate molecules are negatively charged glycosaminoglycan molecules [76]. Thus, some α-helical cationic peptides, e.g., lactoferrin [77], can prevent HSV infections by binding to heparan molecules and blocking virus-receptor interactions [78].

Compared to the above AMPs that target viral receptors on cell surface, some AMPs do not compete with viral glycoproteins for binding to the heparansulphate receptors on cell surface. Instead, these antiviral AMPs can cross the cell membrane and localize in the cytoplasm and organelles, causing changes in the gene expression profile of the host cells, which can help the host defense system fight against viruses or block viral gene expression. For example, NP-1, an AMP from rabbit neutrophils, prevents Vero and CaSki cell lines from infection by herpes simplex viruses type 2 (HSV-2). This AMP stops the viruses by preventing the migration of a major viral protein, VP16, into the nucleus. This viral protein is required to form complexes with the host transcriptional factors to induce the expression of immediate early viral genes, which are required for the virus to defeat the first stage cellular response [79]. Thus, this AMP does not compete with viral particles to bind to the receptor on cell surface but it prevents cell-to-cell spread of viral particles [80].

#### 3.1.2. Antibacterial Peptides

Antibacterial AMPs are the most studied AMPs to date and most of them are cationic AMPs, which target bacterial cell membranes and cause disintegration of the lipid bilayer structure [81,82]. The majority of these AMPs are also amphipathic with both hydrophilic and hydrophobic domains. Such structures provide AMPs the capability to bind to lipid components (hydrophobic region) and phospholipid groups (hydrophilic region) [47].

Interestingly, researchers have demonstrated that some AMPs at low concentrations can kill bacteria without changing the membrane integrity. Instead of directly interacting with the membrane, these AMPs kill bacteria by inhibiting some important pathways inside the cell such as DNA replication and protein synthesis [83]. For example, buforin II can diffuse into cells and bind to DNA and RNA without damaging the cell membrane [84]. Drosocin, pyrrhocoricin, and apidaecin are other examples of such AMPs. These AMPs have 18–20 amino acid residues with an active site for their intracellular target [85,86].

In some cases, certain AMPs have been shown to kill antibiotic resistant bacteria. For example, both nisin (an AMP) and vancomycin (an antibiotic), can block cell wall synthesis. However, a methicillin resistant *Staphylococcus aureus* (MRSA) strain was reported to be resistant to vancomycin, while it is still sensitive to nisin [87].

#### 3.1.3. Antifungal Peptides

Like antibacterial AMPs, antifungal peptides can kill fungi by targeting either the cell wall [88,89] or intracellular components [90]. However, bacterial membrane and fungi cell wall have different contents. For example, chitin is one of the major components of fungal cell walls and some of antifungal peptides are capable of binding to chitin [91,92,93]. Such binding ability helps AMPs to target fungal cells efficiently. Cell wall targeting-antifungal AMPs kill the target cells by disrupting the integrity of fungal membranes [94,95], by increasing permeabilization of the plasma membrane [96], or by forming pores directly [97].

Although the majority of antifungal AMPs have polar and neutral amino acids in their structures, [47] there does not appear to be a clear correlation between the structure of an AMP and the type of cells that it targets. For example, antifungal peptides have members from different structure classes such as α-helical (D-V13K [98] and P18 [99]), extended (indolicin [100]), and β-sheet (defensins [101]).

#### 3.1.4. Antiparasitic Peptides

Antiparasitic peptides are a smaller group compared to other three AMP classes. The first antiparasitic peptide reported is magainin, which is able to kill *Paramecium caudatum* [102]. Later, a synthetic peptide was developed against *Leishmania* parasite [103]. Another example of antiparasitic peptide is cathelicidin, which is able to kill *Caernohabditis elegans* by forming pores in the cell membrane [104]. Even though some parasitic microorganisms are multicellular, the mode of action of antiparasitic peptides is the same as other AMPs. They kill cells by directly interacting with cell membrane [104].

### 3.2. Mechanism of Action

As described above, AMPs kill cells by disrupting membrane integrity (via interaction with negatively charged cell membrane), by inhibiting proteins, DNA and RNA synthesis, or by interacting with certain intracellular targets. All AMPs known by the late-90s are cationic. However, the concept that AMPs need to be cationic was changed later with the discovery of negatively charged AMPs in 1997 [105]. For example maximin-H5 [106] from frog skin and dermicidin [107] secreted from sweat gland tissues of human are both anionic peptides.

Generally an AMP is only effective against one class of microorganisms (e.g., bacteria or fungi) [31]. However, there are exceptions and some AMPs are known to have different modes of action against different types of microorganisms. For example, indolicidin can kill bacteria, fungi, and HIV [69,108]. It exhibits antifungal activities by causing damages to cell membrane [100]. However, it kills *E. coli* by penetrating into the cells and inhibiting DNA synthesis [109]; and it shows anti-HIV activities by inhibiting HIV-integrase [110]. In comparison; some AMPs have the same mode of killing of different cell types. For example, PMAP-23 can kill both fungi and parasites by forming pores in their cell membranes [104,111].

One third of the total proteins of a bacterial cell are associated with the membrane and these proteins have many functions that are critical to the cell including active transport of nutrients, respiration, proton motive force, ATP generation, and intercellular communication [112]. The function of these proteins can be altered with AMP treatment even if complete cell lysis does not occur. Therefore, AMPs’ rapid killing effect does not only come from membrane disruption but can also come from inhibition of these functional proteins.

#### 3.2.1. Membrane-Active AMPs

**Figure 2:**
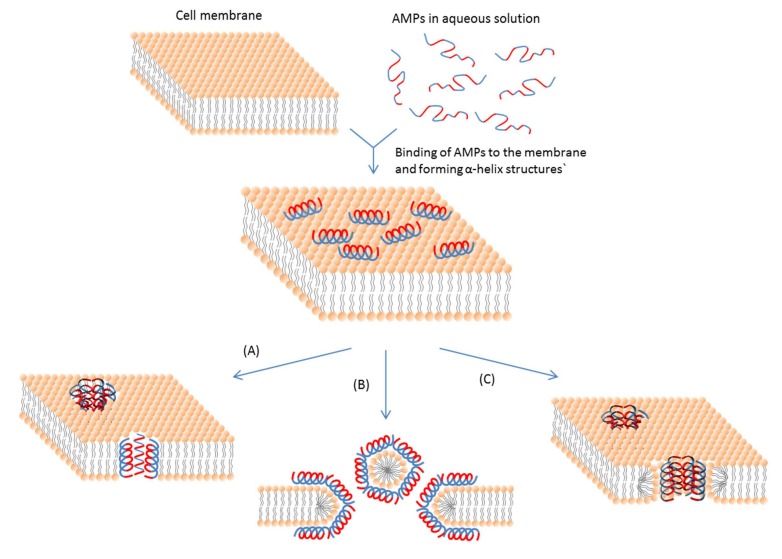
Schematic representation of some action mechanisms of membrane-active AMPs.(**A**) Barrel-Stave model. AMP molecules insert themselves into the membrane perpendicularly. (**B**) Carpet model. Small areas of the membrane are coated with AMP molecules with hydrophobic sides facing inward leaving pores behind in the membrane. (**C**) Toroidal pore model. This model resembles the Barrel-stave model, but AMPs are always in contact with phospholipid head groups of the membrane. The blue color represents the hydrophobic portions of AMPs, while the red color represents the hydrophilic parts of the AMPs.

Even if intracellular targets are involved, an initial cell membrane interaction with peptides is required for the antimicrobial activities of AMPs [113]; and this interaction determines the spectrum of target cells. Most membrane-active AMPs are amphipathic, which means that they have both cationic and hydrophobic faces. This feature ensures the initial electrostatic interaction with the negatively charged cell membrane and the insertion into membrane interior. The actions of AMPs do not stop after this initial interaction. The hydrophobic part of an AMP helps insert the AMP molecule into the cell membrane [114]. So the interaction mainly includes ionic and hydrophobic interactions. These interactions mostly depend on two properties, e.g., cationic state and hydrophobicity of the peptide. The major types of membrane-active AMPs and the mechanisms of their actions are summarized in Table 1 and Figure 2.

**Table 1:** The action mechanisms of membrane-active AMPs.

Interaction model	Mechanism	References
Carpet like (Detergent-like)	The peptide micelle touches the membrane first and coats a small area of the membrane. Then AMP molecules penetrate the lipid bilayer to let pore formation occur leaving holes behind.	[115,116,117]
Membrane thinning	AMPs insert themselves into only one side of the lipid bilayer. It can form a gap between lipid molecules at the chain region. This gap creates a force and pulls the neighboring lipid molecules to fill it.	[118,119,120]
Aggregate	AMPs stick to the membrane parallel to the surface. Then reorientation of AMPs occurs and they insert themselves into the membrane vertically to form sphere-like structures.	[115,121,122,123]
Toroidal pore	AMPs align perpendicularly into the bilayer structure with their hydrophobic regions associated with the center part of the lipid bilayer and their hydrophilic regions facing the pore.	[83,123]
Barrel-stave	Staves are formed first parallel to the cell membrane. Then barrels are formed and AMPs are inserted perpendicularly to the plane of the membrane bilayer.	[82,124,125]

#### 3.2.2. Intracellularly Active AMPs

In early AMP studies, permeabilization of bacterial cell membrane by AMP was thought as the primary mechanism of killing. It was suggested that AMPs should be used at concentrations high enough so that they can kill microorganisms by disrupting the membrane with sufficient channels and pores [126]. However, some AMPs were found to start membrane permeabilization at concentrations lower than their MICs, while others could only do so at concentrations higher than their MICs. The finding that some AMPs can kill their target cells without causing membrane permeabilization suggests that there may be other mechanisms of killing. Recently, intracellularly active AMPs have been shown to interact with targets inside the cells [127,128,129]. For example indolicin was shown to bind to DNA with a preferred sequence [46,130].

Some AMPs can inhibit DNA and protein synthesis [131,132]. One example of this is PR-39, an AMP from pig intestines, which kills bacteria in a non-lytic process by acting like a proteolytic agent and stopping protein and DNA synthesis [133]. Similar to PR-39, indolicin does not lyse cells directly. It enters the cytoplasm and kills bacterial cells by targeting DNA synthesis [109,131]. Also, some human immune system derived AMPs such as tPMP-1 and aHNP-1 inhibit DNA and protein synthesis within an hour after they enter the cells [134]. Apidaecin is another protein synthesis blocking AMP which lacks pore forming ability. This AMP is only effective against Gram-negative bacteria. It is suggested that this AMP is actively transported with a transporter protein and then it blocks protein synthesis with a series of molecular interactions with different targets [135].

Some AMPs can also inhibit proteases of microbes. For example, histatin 5 stops the periodontal tissue destruction by inhibiting a protease from *Bacteriocides gingivalis* [136]; and eNAP-2 has anti-protease activities against microbial serin proteases [137]. Interestingly, there are some intracellular AMPs which can only kill cells at certain growth stages. For example, diptericin is only effective against actively growing bacterial cells, suggesting it may interact with certain specific metabolic pathways during bacterial growth [138,139].

Among these intracellularly active AMPs, some of them have multiple targets. For example seminalplasmin inhibits RNA polymerase and can stop RNA synthesis completely at concentrations lower than many other antibacterial agents [140]. On the other hand, the same AMP can activate an autolysin protein inside the target cells leading to autolysis [141,142].

The finding that AMPs can inhibit intracellular pathways [126,131] suggest that there might be mechanisms of cellular uptake of AMPs. Two such mechanisms have been reported: direct penetration and endocystosis [114]. According to Jones [143], cellular uptake of AMPs can take place through endocytosis, which includes macropinocytosis and receptor mediated endocytosis. In macropinocytosis, the cell membrane folds inward and forms vesicles with the help of dynamin proteins. These vesicles are called macropinosomes and they are like small cells with only a membrane around them [114]. In receptor mediated endocytosis, a part of the membrane is coated with clathrin or caveolin proteins followed by pit formation. Later, these pits bud from the membrane to inner side of the cell and form vesicles [143,144].

## 4. Designing New Synthetic AMPs: Major Factors to Consider

To date, no data have been reported to demonstrate a clear relationship between the structural groups of an AMP and its mode of action, the degree of activity, or the host range. Even the AMPs with very similar structures can have drastically different mechanisms of action and the range of targeted cells [47]. For example, buforin targets DNA and RNA; while magainin 2, an AMP with similar structure, targets the cell membrane causing cell lysis [145,146]. Although a structure-based precise prediction of activity, mode of action, and host range may not be possible, certain general design principles have been proposed by previous studies. The AMP structure is certainly important, while the size, charge, hydrophobicity, amphipathicity, and solubility are all crucial physiochemical properties for their antimicrobial activities and target specificity of AMPs [147]. Changing these features will help to modify the activity and target spectrum of AMPs.

### 4.1. Important Physiochemical Properties of AMPs

#### 4.1.1. Length

The length of an AMP is important to its activity because at least 7–8 amino acids are needed to form amphipathic structures with hydrophobic and hydrophilic faces on opposite sides of a peptide molecule. The size for an AMP to transverse the lipid bilayer of bacteria in the barrel-stave model should be at least 22 amino acids for α-helical AMPs, while eight amino acids are needed for β-sheet AMPs [148]. Besides the effects of length on its 3D structure and mode of action, the length of an AMP may also affect its cytotoxicity. For example, a shortened melittin with 15 residues at its C-terminal [149] and a shorter derivative of HP(2-20) [150] exhibited at least 300 times less toxicity to rat erythrocytes and human erythrocytes, respectively, compared to their original forms. Therefore the length of AMP should be taken into consideration when designing new synthetic peptides with low toxicity.

#### 4.1.2. Net Charge

The net charge of known AMPs, which is the sum of all charges of ionizable groups of the peptide, varies from negative to positive and it is the main factor for the initial interaction with negatively charged cell membranes. By changing the net charge of an AMP, its antimicrobial and hemolytic activities can be altered to achieve selective killing of microbes with no or minimized effects on host cells. For example, increasing positive net charge of V13K from +8 to +9 resulted in higher hemolytic activity, while decreasing the net charge to lower than +4 abolished its activity against *P. aeruginosa* [98].

#### 4.1.3. Helicity

Helicity represents the ability of an AMP to form spin structure. It is less important for the activity of an AMP compared to other factors discussed above. However, it is important for determining the toxicity on eukaryotic cells [6]. Reducing helicity by incorporating d-amino acids into the primary sequence has been shown to lower the hemolytic effect, while the antimicrobial effect was retained [151]. For example, Papo *et al.* [54] modified some α-helical peptides by replacing 35% of the l-amino acids with d-amino acids and found that this modification eliminated the hemolytic activity. Besides, these new synthetic AMPs are not sensitive to proteases. Therefore, incorporating d-amino acids to change helicity is a useful strategy for designing new synthetic peptides with less hemolytic activity and enhanced stability against proteolytic cleavage. Another important factor associated with the helicity of AMP is the helix propensity of each amino acid in the primary sequence. For example, proline and glycine have lower helix-forming propensities compared to other amino acids [152]. Thus, these residues are not preferred when designing α-helical AMPs. In addition, peptides should be flexible enough to change their conformation during the membrane insertion process [47].

#### 4.1.4. Hydrophobicity

Hydrophobicity has also been shown to influence the activity and selectivity of AMP molecules. Almost 50% of amino acids in the primary sequence of natural AMPs are hydrophobic residues [147]. In most cases, increase in hydrophobicity on the positively charged side of an AMP below a threshold can increase its antimicrobial activity [6], while decreasing hydrophobicity can reduce antimicrobial activity [153]. There appears to be an optimal hydrophobicity for each AMP, beyond which its activity decreases rapidly [129]. Therefore, when designing new synthetic peptides, the hydrophobicity should be selected within an optimal window. Some previous studies have shown that hydrophobicity is also critical for determining the range of target cells of an AMP. Increasing the hydrophobicity of an AMP can change the range of targets [154,155]. For example, magainin is an AMP that is only effective against Gram-negative bacteria. However, some synthetic analogs with higher hydrophobicity can also kill some Gram-positive bacteria and eukaryotic cells [156].

#### 4.1.5. Amphipathicity

Amphipathicity is another important property of AMPs to ensure their activity and interaction with microbial membranes. Fernandes-Vidal *et al.* [157] showed that amphipathicity is more important than hydrophobicity for binding to microbial membranes. Because amphipathicity of AMPs is required for a strong partition into the membrane interface, priority should be given to the amphipathic structure when designing synthetic AMPs for specific target cells.

#### 4.1.6. Solubility

Since AMPs should act on or enter through lipid membranes, they need to be soluble in aqueous environments. If AMP molecules aggregate, it will lose its ability to interact with the cell membrane. For example, a hybrid synthetic AMP composed of cecropin and melittin has a tendency to form dimers. Substituting a Lys residue on the non-polar face of this hybrid AMP prevents dimerization and leads to reduced hemolytic activity. Losing dimerization ability makes this AMP more effective for its incorporation into microbial membranes [158]. This example demonstrates the importance of solubility and the value of structural optimization.

### 4.2. The Relationship between Physiochemical Properties of AMPs

As discussed above, many factors affect the activities of AMPs and some interactions exist between these factors. In AMP design, these properties need to be considered together since changing one of these parameters to get a desired modification of an AMP may alter other parameters. Even a simple change in primary sequence can affect many other physicochemical parameters which are often vital for the activity of an AMP and the range of target cells [159]. Predicting the results of an AMP modification or the function of a synthetic peptide beforehand is still an unmet challenge. Application of molecular simulation to analyze the details of the folding of AMP molecules and interaction with target cells [160,161] may be a promising approach to improve current trial and error methods.

### 4.3. AMP Modifications

While most of AMPs are directly synthesized in their active forms, post-translational modification of certain AMPs is necessary for their functions. Naturally forming AMPs are processed with different post-translational modifications such as phosphorylation [162], addition of d-amino acids [163,164], methylation [165], amidation [166], glycosylation [167], formation of disulphide linkage [168], and proteolytic cleavage [24,169]. In some cases, these posttranslational modifications might be important for designing new synthetic AMPs. Even though recombinant cell systems can be used to produce these synthetic peptides with post-translational modifications, incorporation of unnatural amino acids may require chemical synthesis [60].

#### 4.3.1. Modification of AMPs with Covalent Bonds

Covalent modification can have profound effects on the structure and function of an AMP. Even a single disulfide bond can change the antimicrobial effect of an AMP. For example, protegrin missing a disulphide bond becomes inactive against HSV [170]; while adding disulphide bond in sakacin P resulted in higher antimicrobial activities [44]. In another study, a disulfide bond was added in CP-11, a derivative of indolicidin [171], and a trp-trp cross-link was added in indolicin [172]. These modified structures of indolicidins showed higher protease stability with no change in antimicrobial activity. However increase in stability does not always lead to better AMPs. For example, Houston *et al.* [173] introduced a covalent bond to form a lactam bridge between Gln and Lys residues in two α-helical AMPs, e.g., cecropin and mellitin. This modification helped AMPs to form more stable α-helix structures but decreased the antimicrobial activity of both.

#### 4.3.2. Modification of AMPs by Changing Amino Acid Content

Alteration of amino acid content is one of the most studied strategies of AMP modification. Most of these studies focus on certain amino acids since their physiological characteristics play important roles in the activity and target spectrum of AMPs. For example proline content in the primary sequence of an AMP has been found to affect its ability to penetrate cell membranes. Higher proline content reduces the capability of CP26 to permeablize *E. coli* cell membrane [174]. This effect might be because of proline’s low propensity to form α-helical structures. Thus changes in the proline content may lead to alterations of α-helical posture of an AMP.

Changing amino acid content can also affect cytotoxicity. In a study by Nell *et al*. [175], LL37, a human AMP, was modified by removing neutral amino acids Asn and Gln, and adding more positively charged residues (two Arg units) into the primary sequence. The new synthetic peptide showed less cytotoxic effects on eukaryotic cells. This peptide was named P60.4 and has been successfully used in nasal applications against MRSA [176]. Another strategy to improve AMP stability is to include, d-amino acids in the sequence because they are more tolerant to proteases [177,178].

#### 4.3.3. Modification of AMPs by Amidation

With new developments in peptide synthesis, it is possible to incorporate special chemical groups or unnatural molecules into AMPs. One of these modifications is the addition of amide groups at the end of the peptides. In 2011, Kim *et al.* [179] modified PMAP-23 with amidation at the carboxyl end and found that this derivative of PMAP-23 orients perpendicularly inside the bacterial membrane while original PMAP-23 orients parallel to the membrane. This modification resulted in almost 10 fold higher cellular uptake, faster interaction with Gram-negative bacteria cell membrane, and deeper insertion into the inner membrane than the original PMAP-23. This carboxyl-end amidated synthetic peptide also showed better membrane-permeabilization in liposome release tests [179]. Therefore amidation of carboxyl end has good potential to improve the function of synthetic AMPs.

C-terminal modifications can also affect the stability of AMPs. In a previous study by Berthold *et al.* [180], the C-terminal amide group of Api88 was replaced by a free acid. This modification did not change its antimicrobial activity, but resulted in a 15 times more stable Api88 derivative against proteases in blood serum. Replacing Arg-17 of this AMP with l-ornithine or l-homoarginine gave 35 times higher proteolytic stability than the original Api88. However, the latter modification decreased the antimicrobial activity by eight fold [180].

#### 4.3.4. Modification of AMPs with Unnatural Amino Acids

A number of studies on synthetic peptides have attempted to incorporate unnatural amino acids into the primary sequence [99,181,182]. β-didehydrophenylalanine is an unnatural amino acids and is used to provide better folding properties for AMPs [181]. It is widely used in medicinal chemistry to alter the native bioactive AMPs [183]. Incorporation of β-didehydrophenylalanine in the primary sequence of VS1 resulted in higher stability against proteases. Researchers have also been able to introduce antifungal activities to some AMPs by incorporating undecanoic acid and palmitic acid into their primary sequence [99,184].

#### 4.3.5. Modification of AMPs with Computer-Assisted Methods

The use of computer-assisted methods in AMP research has been increasing significantly [185,186,187,188,189,190]. Estimating the structure of an AMP based on its primary sequence [191], then predicting potential mechanism of action and activity is becoming easier with the help of computational approaches [192]. These types of artificial AMP design strategies hold potential for developing new synthetic peptides against antibiotic-resistant superbugs [190]. Several databases about AMPs have been created and can be accessed to compare currently available AMPs. One of the latest AMP databases, LAMP (linking antimicrobial peptides), currently has 3904 natural and 1643 synthetic peptides [19].

### 4.4. New AMP Design by Homology Modeling

Most studies about AMPs to date are inspired by natural AMPs. For example, Tossi *et al.* [193] designed some synthetic peptides by identifying the common amphipathic structure of 87 different natural α-helical AMPs. These natural AMPs are composed mainly of cecropins, magainins, brevinins, and cathelicidin peptides sourced from insects, amphibian, and mammals. This synthetic peptide study focused on the first 20 amino acids in each sequence because the N-terminal region was shown to be necessary for antimicrobial activities [194,195]. The synthetic peptides designed based on this strategy are able to transform into α-helical structures from random structures with the addition of trifluoroethanol in aqueous environments. These synthetic AMPs exhibited antimicrobial activities against Gram-positive and Gram-negative bacteria, including some drug resistant strains. In addition, these synthetic AMPs showed low toxicity to some eukaryotic cell lines [193].

Designing synthetic AMPs by homology modeling within the same class might also provide a better understanding of activity-structure relationship. Important elements from the same AMP class may be identified using this approach to help design better molecules. Storici *et al.* [195] showed that 20 residues (named PMAP-36) of an antibacterial peptide from pig bone marrow cells are sufficient for related antibacterial activity. The AMP with these 20 residues was chemically synthesized and showed the capability to form α-helix in the presence of trifluoroethanol. This short synthetic peptide was found to induce permeabilization of the inner membrane of *E. coli* ML35 at concentrations lower than 50 µM; while even at 100 µM it did not cause any permeabilization to human erythrocytes [195]. In another study of homology modeling, arenicin, protegrin, and thanatin were used as templates to generate three synthetic peptides: AMP72, AMP126 and AMP2041. These new synthetic AMPs showed lower cytotoxicity compared to the original AMPs and exhibited dose dependent antimicrobial activities (0.17 to 10.12 μM) against Gram-negative bacteria [196].

It is also possible to broaden the target spectrum of an AMP by homology modeling. For example, normally lactoferrampins are not effective against *E. coli* O157. A conserved sequence, which corresponds to an α-helical region, among these AMPs was found, by aligning multiple sequences with ClustalW analysis. This common region was modified by inserting GKLI sequence into its primary sequence, and the new synthetic peptide showed activities against *E. coli* O157 with a more stable structure compared to other lactoferrampins [197].

## 5. New Targets of AMPs: Biofilms, Persister Cells, and Drug Resistance Bacteria

Because AMPs can directly target bacterial cells, they have potential to control antibiotic tolerant cells. Here we review some recent work on biofilms and persister cells. Biofilms are immobile bacterial populations attached to surfaces such as human tissues and medical implants. Biofilm formation on implant surfaces is a serious problem since every year more than $3 billion is spent to treat implant-associated biofilm infections in the U.S. alone [198]. With cells protected by an extracellular matrix, biofilms are highly tolerant to antimicrobials [199] and are a major cause of chronic infections; e.g., approximately 80% of human bacterial infections are associated with biofilms [200]. In addition to the protection by the extracellular matrix [201], biofilm associated antibiotic resistance is also attributed to the slow growth of biofilm cells [202]. Even though some antibiotics have been shown to effectively penetrate biofilm matrix [203], they are not effective against these slowly growing cells, especially the dormant subpopulation known as persister cells [204,205,206]. Since most AMPs target cell membrane, they may be more effective against these dormant cells compared to antibiotics.

### 5.1. Biofilm Control

The first obstacle of using AMPs against biofilms is the possible electrostatic interaction between cationic peptides and negatively charged biofilm matrix [207]. Such interactions may retard or prevent AMPs from reaching biofilm cells. Previous studies have investigated the effects of some AMPs on biofilm inhibition and killing of bacterial cells in established biofilms. The second type of study is especially important since treatment of mature biofilms is highly challenging [205]. In a study by Singh *et al.* [208], lactoferrin was found to block biofilm formation of *P. aeroginosa* at concentrations lower than those required to kill the planktonic cells. Also, LL-37, a human cathelicidin AMP, was shown to prevent *P. aeruginosa* biofilm formation at the concentration of 0.5 µg/mL, which is below its MIC (64 µg/mL). This AMP also showed activity against preformed (2-days old) *P. aeruginosa* biofilms; e.g., it reduced the biofilm thickness by 60% and destroyed microcolony structures of the treated biofilms [209]. In another study, a derivative of LL-37 was found effective against both Gram-positive and Gram-negative bacteria. Despite its weak antimicrobial activity against planktonic cells, this AMP inhibited biofilm formation of *P. aeruginosa*, *Burkholderia cenocepacia*, and *Listeria monocytogenes* with more than 50% reduction in biofilm mass compared to untreated controls [210]. The same study showed that this inhibition is due to decrease in swarming and swimming motilities, increase in twitching motility, and repression of some biofilm genes.

In addition to free AMPs, surface coating with AMPs has also been pursued since surface modifications with AMPs might help reduce device associated infections [211,212,213,214]. Many AMPs have been tested for their inhibitory effects on biofilm formation on implant surfaces. For example, Tet-20, a synthetic peptide (KRWRIRVRVIRKC), tethered on an implant surface exhibited broad antimicrobial activities both *in vivo* (rats) and *in vitro*. It is able to stop biofilm formation and appears to be non-toxic to eukaryotic cells [211]. In another study, histatin 5 and lactoferrin were used to coat Ti surfaces covered with an anchor peptide minTBP (RKLPDAP), which helps binding of AMP to Ti surfaces. The conjugates of both AMPs resulted in higher binding efficiency to Ti surfaces than AMPs alone and *Porphyromonas gingivalis* showed less ATP activity and reduced biofilm formation on coated surfaces [215].

In addition to naturally existing AMPs, some synthetic AMPs were also used to treat biofilms. A synthetic histatin analogue dhvar4 was tested against oral flora on hydroxyapatite disks and this AMP reduced the number of viable biofilm cells by 1.5 log compared to the control [216]. MUC7, a native saliva AMP from humans, and its modified forms, MUC17 12-mer-L and 20-mer, showed inhibitory effects on *S. mutans* biofilm formation [217]. In another study, a derivative of LL-37 which is an AMP from human innate immune system, cleared *P. sinusitis* biofilms *in vivo* (New Zealand rabbits). However, it also led to some toxicity and proinflammation in the sinuses [218].

As discussed above, the extracellular matrix of a biofilm is thought to form a diffusion barrier against certain AMPs [199]. It is known that this negatively charged barrier protects the cells inside from positively charged antimicrobial agents and the alginate in biofilm matrix can reduce the diffusion of antimicrobial agents [219]. Thus, it is important to obtain AMPs that can diffuse into biofilms and kill biofilm cells. Recently a synthetic peptide, (RW)_4D_ dendrimer [220] was demonstrated to inhibit planktonic growth and biofilm formation of *E. coli* dose dependently. This AMP inhibited biofilm formation by 93.5% at 40 µM. This dendrimer did not detach preformed biofilms, but was able to kill most of the cells residing in mature biofilms dose dependently [221]. Later, (RW)_n_-NH_2_ based AMPs with different chain length (where n = 2, 3, and 4) were compared for their effects on *E. coli* RP437 biofilms. The chain length was found to be important to the activity of these peptides. Longer peptides, (RW)_3_-NH_2_ and (RW)_4_-NH_2_, showed significant inhibition of planktonic growth (36% reduction in growth rate) while a shorter peptides (RW)_2_-NH_2_ did not cause a clear inhibition at concentrations up to 200 µM. This length-activity relation was also found for biofilm inhibition. *E. coli* biofilm surface coverage and the viability of biofilm cells were reduced significantly by the longer peptides (95% inhibition of biofilm growth by 200 µM (RW)_3_-NH_2_ and 84.4% inhibition of biofilm growth by 200 µM (RW)_4_-NH_2_).Preformed biofilms were also tested with these peptides. However, the treatment of preformed biofilms with these peptides did not show the same length-activity relationship. Interestingly, 200 µM (RW)_3_-NH_2_ showed significant killing of biofilm cells while 200 µM (RW)_4_-NH_2_ showed strong biofilm dispersion (91.5% reduction in biofilm surface coverage at 200 µM) with no apparent killing effect on biofilm cells. Although 200 µM (RW)_4_-NH_2_ did not kill biofilm cells directly, the detached biofilm cells were killed by this peptide effectively [222].

AMPs have also been tested against the biofilms of drug resistant bacteria. In a study by Okuda *et al.* [223], nisin A and lacticin Q were tested against mature biofilms of a MRSA strain, *S. aureus* MR23. Nisin A at 40 µM was found to kill more than 95% biofilm cells while lacticin Q at 80 µM killed around 90% of the biofilm cells. In another study, GL13K derived from human parotid secretory protein (PSP) killed 99.9% of 24 hour biofilm cells of *P. aeruginosa* when it was added at 100 µg/mL for a two hour treatment [224].

NRC-16, a synthetic peptide, was tested against biofilm formation of three *P. aeruginosa* strains and compared with antibiotics such as ampicillin, chloramphenicol, and ciprofloxacin. NRC-16 showed biofilm inhibition at 8 µg/mL, which is 64 fold less than the antibiotic concentrations required to kill these *P. aeruginosa* strains [225].

There are also some AMPs that can sensitize biofilm cells to other antimicrobial agents. For example, lactoferrin does not kill *S. epidermidis* or affect its growth. However treatment of *S. epidermidis* biofilms on contact lenses with lactoferrin and vancomycin together showed a 2 fold decrease in both MBC (minimal bactericidal concentration) and MIC of biofilm cells compared to the treatment with vancomycin alone [226].

### 5.2. Persister Control

Persisters cells can be found in almost any microbial populations. These cells are dormant phenotypic variants and are highly tolerant to antibiotics [227]. However, membrane integrity is essential for the survival of bacteria irrespective of the metabolic stage of the cell and cell membrane is a major target of AMPs. Thus, AMPs may have good potential to kill persister cells. In a recent study, a synthetic cationic peptide, (RW)_4_-NH_2_, was found to kill more than 99% of *E. coli* HM22 persister cells in planktonic culture. Besides, this synthetic peptide reduced the number of persister cells in mature biofilms by up to 98% at 40 µM. More interestingly, the combination of this peptide with oflaxacin (5 µg/mL) resulted in complete eradication of viable cells in *E. coli* HM22 biofilms including persister cells [228]. Thus, the combination of conventional antibiotics with AMPs may offer a synergy to control drug tolerant infections.

## 6. Resistance to Antimicrobial Peptides

**Figure 3:**
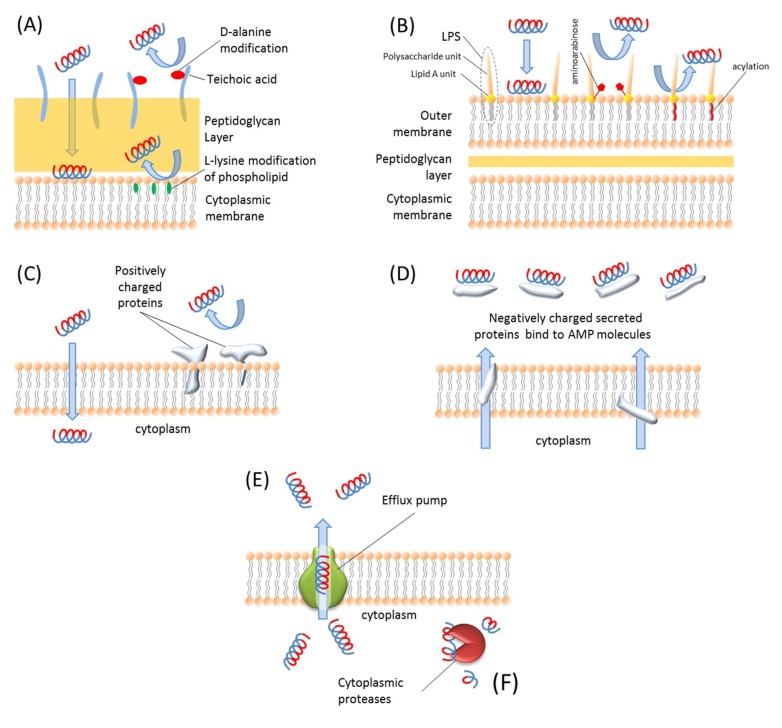
Schematic representation of AMP resistance mechanisms. (**A**) Gram-positive bacteria resist AMPs via teichoic acid modification of LPS molecules and l-lysine modification of phospholipids. (**B**) Gram-negative bacteria resist AMPs by modifying LPS molecules with aminoarabinose or acylation of Lipid A unit of LPS molecules. (**C**) Bacteria express some positively charged proteins and integrate them in the membrane so positive charges repulse each other and bacteria can resist such AMPs. (**D**) Bacteria produce negatively charged proteins and secrete them into extracellular environment to bind and block AMPs. (**E**) The intracellular AMPs are extruded by efflux pumps. (**F**) The AMPs inside the cell are degraded by proteases.

There are mainly two different types of resistance mechanisms against AMPs: constitutive resistance and inducible resistance [229]. The inducible resistance mechanisms include substitution [230], modification [231], and acylation [232] of the membrane molecules, activation of some proteolytic enzymes [233] and efflux pumps [234], and modifications of intracellular targets [235]. The constitutive resistance mechanisms include electrostatic shielding [236], changes in membrane potential during different stages of cell growth [237], and biofilm formation [229]. These resistance mechanisms are illustrated in Figure 3.

For example the activity of some AMPs against *S. aureus* can be inhibited by adhesin molecules on the cell surface of this bacterium. These adhesin molecules are polymeric substances and stay on the cell surface after secretion [238]. Since adhesin is a positively charged polymer, it can form a repulsive barrier against positively charged AMPs. *Salmonella typhimurium* also has a membrane bound lipid A modification system, which defends themselves against AMPs from the host [239]. In this system, PhoQ is a membrane bound sensor kinase and PhoP is intracellular response regulator. PhoQ is activated in the presence of high level positive charges outside the cells. It then phosphorylates the PhoP causing up-regulation of some genes including those related to AMP resistance. This system is not active when the extracellular level of Ca^2+^, Mg^2+^, or Mn^2+^ ions is low since divalent cations interact with PhoQ and change its conformation [63].

Although bacteria have diverse mechanisms for resistance to AMPs, it is encouraging to notice that the general lipid bilayer structure of bacterial membranes makes it hard to develop a complete resistance against AMPs. Also, the resistance against AMPs reported to date is not as strong as those against antibiotics and it only covers a limited number of AMPs.

## 7. Conclusions

The urgent need to obtain new antimicrobials has been driving AMP research. With rapid growth in related knowledge and lead compounds, more AMPs may enter clinical tests and treatment in the near feature. However, infection control by AMP is still hindered by several challenges including low specificity, high manufacturer cost, potential toxicity to animal cells, and lack of a robust guideline for rational design.

As we have seen from synthetic and modified AMP studies, it is easy to change characteristics of an AMP with even small modifications. However, predicting the results of these changes is still challenging. Thus, there is a need to understand the effects of structural modifications on the physiochemical characteristics of AMPs as well as their target spectrum and activity. Recently, these types of studies have been increasing and computational approaches have been involved in AMP research. These efforts will help to better understand the mode of action of AMPs and predict their activities.

Another understudied area is using AMPs to control antibiotic resistant bacteria, biofilms, and persisters. These targets are highly resistant to traditional antibiotics and play important roles in infections. Since AMPs target cell membrane, they have good potential in such applications. On the other hand, because AMPs have not been well studied for biofilm and persister control, there might be some existing natural AMPs that are effective against these targets with potential synergy with antibiotics. Applying AMPs with biofilm matrix degrading enzymes might also be a good strategy to eliminate biofilms. Further development in this area and AMP research in general will benefit from close collaboration of different disciplines and new tools that can decipher the structure-function relationship and more efficiently synthesize and modify AMP molecules.
